# Parkinson’s Disease Progression: Implicit Acquisition, Cognitive and Motor Impairments, and Medication Effects

**DOI:** 10.3389/fnint.2012.00056

**Published:** 2012-08-10

**Authors:** Rodrigo Pavão, André Frazão Helene, Gilberto Fernando Xavier

**Affiliations:** ^1^Biosciences Institute, University of São PauloSão Paulo, Brazil; ^2^Brain Institute, Federal University of Rio Grande do NorteNatal, Brazil

**Keywords:** PD, progression, cognitive functions, levodopa, modeling

## Abstract

Parkinson’s disease (PD) symptoms have been collectively ascribed to malfunctioning of dopamine-related nigro-striatal and cortico-striatal loops. However, some doubts about this proposition are raised by controversies about the temporal progression of the impairments, and whether they are concomitant or not. The present study consists of a systematic revision of literature data on both functional PD impairments and dopaminergic medication effects in order to draw a coherent picture about the disease progression. It was done in terms of an explanatory model for the disruption of implicit knowledge acquisition, motor and cognitive impairments, and the effects of dopaminergic medication on these functions. Cognitive impairments arise at early stages of PD and stabilizes while disruption of implicit knowledge acquisition and motor impairments, are still in progression; additionally, dopaminergic medication reduces motor impairments and increases disruption of implicit knowledge acquisition. Since this model revealed consistency and plausibility when confronted with data of others studies not included in model’s formulation, it may turn out to be a useful tool for understanding the multifaceted characteristics of PD.

## Introduction

Parkinson’s disease (PD) is a progressive neurodegenerative condition that has typically been considered to be a motor disorder associated to basal ganglia dysfunction (Marsden, 1982). The main features of PD (i.e., akinesia or bradykinesia, rigidity, and tremor) are mainly related to dysfunction of the motor circuit, involving basal ganglia, thalamus, and motor cortex (Rodriguez-Oroz et al., 2009). Additionally to motricity, the basal ganglia networks are now known to be anatomically and physiologically associated to learning and working memory (Sawamoto et al., 2008; Marklund et al., 2009). The association of motor (performance of skilled movements), implicit learning (degree of improvement by repetitive performance of a task, without declarative knowledge about the reasons for this improvement) and cognitive symptoms (dependent of working memory and attention) along progression of PD are a matter of debate.

Muslimovic et al. (2007) proposed that cognitive impairments are independent of disruption of implicit knowledge acquisition. Similarly, Cooper et al. (1991) reported weak correlations between cognitive and motor symptoms in patients at early stages of PD tested under no effects of dopaminergic medication. On the other hand, Fama and Sullivan (2002) showed strong correlations between motor impairments and disruption of implicit knowledge acquisition in patients tested under effect of dopaminergic medication, and Vandenbossche et al. (2009) reported correlations between motor and cognitive impairments in patients scored at the same Hoehn and Yahr (1967) stages, when tested under effect of dopaminergic medication. Additionally, Pavão et al. (unpublished) showed positive correlation between motor, implicit acquisition, and motor impairments in patients tested under no effect of dopaminergic medication.

These seemingly inconsistent findings may be ascribed to non-linear relationships among these impairments, and that these functions are differently influenced by dopaminergic medication. For formalizing this conception, we propose a single unifying model for explaining the progression of PD, supported by literature data relative to patients and healthy volunteers tested both with and without the effects of dopaminergic medication.

## The Proposal of a Unifying Model

Assuming that the impairments in PD progress in three major domains, including motor performance, implicit knowledge acquisition, and general cognitive changes, and that each of these domains suffer distinct influences of dopaminergic medication administration, we analyzed findings of different studies reporting on the progression of these impairments in PD patients, as well on the effects of dopaminergic medication on the performance of healthy subjects submitted to these tests.

**Figure 1:**
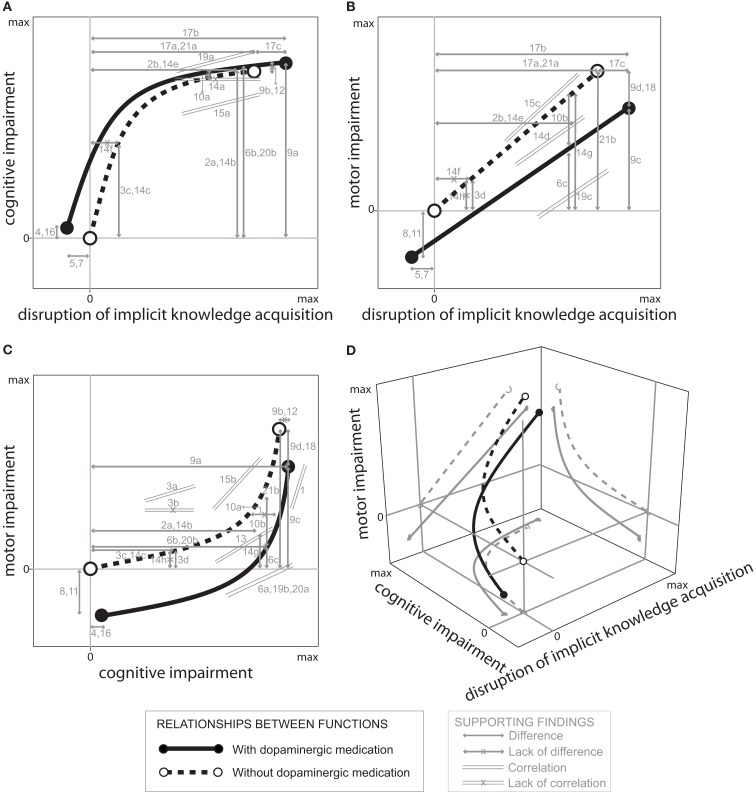
**Model showing the relationships between cognitive impairments as a function of disruption of implicit knowledge acquisition (general findings better adjusted to an arc-tangent function) (A), motor impairment as a function of disruption of implicit knowledge acquisition (general findings better adjusted to a linear function) (B), motor impairment as a function of cognitive impairment (general findings congruent with the other functions) (C), and an unified tri-dimensional model expressing the interaction between motor impairment, disruption of implicit knowledge acquisition, and cognitive impairment (D), in healthy volunteers (represented by the lines corresponding to the “zero” cognitive impairment, the “zero” disruption of acquisition of implicit knowledge and the “zero” motor impairment, tested without dopaminergic medication) and patients with PD tested with and without dopaminergic medication effects along disease progression up to a maximum (max) of disruption**. The studies that lend support to this model are indicated by identification codes (see below) and their results (i.e., differences, lack of differences, and correlations) are represented by gray lines with their extreme points corresponding to compared samples. Identification codes: Caviness et al. (2007) (1), Cools et al. (1984) (2a, 2b), Cooper et al. (1991) (3a, 3b, 3c, 3d), Delaveau et al. (2005) (4), de Vries et al. (2010) (5), Fama and Sullivan (2002) (6a, 6b, 6c), Floel et al. (2005) (7), Floel et al. (2008) (8), Girotti et al. (1986) (9a, 9b, 9c, 9d), Growdon et al. (1998) (10a, 10b), Hasbroucq et al. (2003) (11), Morrison et al. (2004) (12), Mortimer et al. (1982) (13), Muslimovic et al. (2007) (14a, 14b, 14c, 14d, 14e, 14f, 14g, 14h), Pavão et al. (unpublished) (15a, 15b, 15c), Sabbe et al. (2004) (16), Seo et al. (2010) (17a, 17b, 17c), Stocchi et al. (2005) (18), Vandenbossche et al. (2009) (19a, 19b, 19c), Verbaan et al. (2007) (20a, 20b), Wilkinson and Jahanshahi (2007) (21a, 21b; see Table S1 in Supplementary Material for a summary of the original data).

This analysis revealed major trends (see Table S1 in Supplementary Material) and provided information to propose a unifying model aiming at explaining the relationships among these three domains of disease progression, and how dopaminergic medication affects the corresponding test results [Figure 1 – numbers and letters (identification codes) identify studies which lend support to this model and also identify the references included in Table S1 in Supplementary Material]. Although the model takes into account interactions among all three domains, it is described by analyzing the interactions between pairs of domains to facilitate understanding.

### Disruption of implicit knowledge acquisition and cognitive impairments

Figure 1A represents the increase in general cognitive impairments as a function of disruption of implicit knowledge acquisition; while a leftward shift of the curve represents better acquisition of implicit knowledge, an upward shift indicates poorer cognitive performance. This figure shows that there is a rapid initial increase in cognitive impairment in PD when disruption of implicit knowledge acquisition remains almost absent; as the disease further progresses, this is followed by a much slower increase in cognitive impairments associated with acceleration of the disruption of implicit knowledge acquisition, assessed either with or without dopaminergic medication effects. In addition, while dopaminergic medication shifts the curve slightly leftwards and upwards in both healthy volunteers and patients at early stages of PD, it extends the curve rightwards in patients at advanced stages of PD.

These relationships are supported by data from different studies indicated by the identification codes shown in Figure 1 and Table S1 in Supplementary Material. As seen above, Muslimovic et al. (2007) showed that disruption of implicit knowledge acquisition and cognitive impairments do not correlate with each other along PD progression (Figure 1A, identification code 14a indicating lack of significant correlation for PD patients at advanced stages). Differently, the study by Vandenbossche et al., 2009; Figure 1A, identification code 19a) and Pavão et al. (unpublished; identification code 15a) revealed consistent correlations between performances of PD patients in tasks evaluating these functions.

This apparent conflict seems to be related to the inclusion of patients at different disease stages. The study by Muslimovic et al. (2007) evaluated some PD patients at early disease stages but mostly patients at advanced disease stages; in contrast, the studies by Vandenbossche et al. (2009), Pavão et al. (unpublished), and Mortimer et al. (1982) included patients varying from early to advanced stages (see Table S1 in Supplementary Material).

As indicated in Figure 1A, at early stages of PD cognitive impairments are associated with relatively preserved implicit knowledge acquisition. Cooper et al. (1991) and Muslimovic et al. (2007) data lend support to this conclusion: the identification codes 3c and 14c (see Table S1 in Supplementary Material; Figure 1A), shows significant cognitive impairments by non-medicated PD patients at early stages of the disease compared to healthy subjects (represented in Figure 1 as “zero” impairments); identification code 14f (see Table S1 in Supplementary Material; Figure 1A) shows lack of significant disruption of implicit knowledge acquisition by non-medicated PD patients at early stages of the disease relative to healthy subjects).

At advanced stages of PD cognitive impairments have already reached a plateau, which is not changed by dopaminergic medication (Figure 1A, identification codes 9b and 12; Girotti et al., 1986; Morrison et al., 2004; see Table S1 in Supplementary Material) while disruption of implicit knowledge acquisition is still progressing; thus, assessments involving only patients at extreme stages of PD should reveal no correlations (Figure 1A, 14a; Muslimovic et al., 2007) associated with substantial disruption relative to healthy subjects both in cognitive (Figure 1A, identification codes 2a, 6b, 9a, 14b, and 20b; Cools et al., 1984; Girotti et al., 1986; Fama and Sullivan, 2002; Muslimovic et al., 2007; Verbaan et al., 2007), and implicit knowledge acquisition tasks (Figure 1A, identification codes 2b, 14e, 17a, 17b, and 21a; Cools et al., 1984; Muslimovic et al., 2007; Wilkinson and Jahanshahi, 2007; Seo et al., 2010). In contrast, as seen above, assessments of patients at diverse disease stages, like those participating in Pavão et al. (unpublished) and Vandenbossche et al. (2009), revealed significant correlations between these two impairments (Figure 1A, identification codes 15a and 19a).

The model also takes into account that healthy volunteers subjected to dopaminergic medication exhibit small but significant cognitive impairments (Sabbe et al., 2004; Delaveau et al., 2005; Figure 1A, identification codes 4 and 16) and small but significant improvements in implicit knowledge acquisition (Floel et al., 2005; de Vries et al., 2010; Figure 1A, identification codes 5 and 7; see Table S1 in Supplementary Material).

In contrast, PD patients tested without the effects of dopaminergic medication do not exhibit changes in cognitive function when compared to PD patients tested under dopaminergic medication effects (Girotti et al., 1986; Growdon et al., 1998; Morrison et al., 2004; Figure 1A, 9b, 10a, and 12). In addition, PD patients at advanced stages of the disease tested without the effects of dopaminergic medication exhibit small but significant disruption of implicit knowledge acquisition relative to patients at corresponding stages tested under dopaminergic medication effects (Seo et al., 2010; Figure 1A, identification code 17c; see Table S1 in Supplementary Material).

### Disruption in acquisition of knowledge implicit and motor impairment

The relationship between disruption of implicit knowledge acquisition and motor impairment was assumed to be linear (Figure 1B), thus capturing evidence of positive correlations between these functions (Muslimovic et al., 2007; Vandenbossche et al., 2009; Pavão et al., unpublished). The model represents the well-known benefit of dopaminergic medication reducing motor impairments (e.g., Girotti et al., 1986; Growdon et al., 1998; Stocchi et al., 2005; Figure 1B, identification codes 9d, 10b, and 18; see Table S1 in Supplementary Material) and dopaminergic medication-induced worsening of disruption of implicit knowledge acquisition (e.g., Seo et al., 2010; Figure 1B, 17c). In addition, the model also incorporates evidence that healthy volunteers subjected to dopaminergic medication exhibit improvement in both motor (Hasbroucq et al., 2003; Floel et al., 2008; Figure 1B, 8 and 11) and implicit knowledge acquisition (Floel et al., 2005; de Vries et al., 2010; Figure 1B, 5 and 7).

The motor and implicit knowledge acquisition functions are thought to be closely related in PD. Muslimovic et al. (2007) reported significant correlations of performance in tasks evaluating disruption in implicit knowledge acquisition and motor impairments (Figure 1B, 14d). Cooper et al. (1991) showed that patients at early stages of PD tested without the effects of dopaminergic medication exhibited poorer performance in a motor task when compared to healthy subjects in a finger movement test (Figure 1B, 3d).

A different effect was reported by Muslimovic et al. (2007) for patients at early stages of PD tested without the effects of dopaminergic medication both in a motor (Figure 1B, 14h) and implicit knowledge acquisition tasks (Figure 1B, 14f). However, when these authors included data from patients at advanced stages of PD tested with the effects of dopaminergic medication, the whole group exhibited poorer motor performance and implicit knowledge acquisition when compared to healthy volunteers (Figure 1B, 14g and 14e).

Similar findings either with or without dopaminergic medication were reported by Cools et al. (1984), Seo et al. (2010), and Wilkinson and Jahanshahi (2007), relative to implicit knowledge acquisition (Figure 1B, 2b, 17a, 17b, and 21a), and by Fama and Sullivan (2002), Girotti et al. (1986), and Wilkinson and Jahanshahi (2007), relative to motor performance in patients receiving dopaminergic medication compared to healthy subjects (Figure 1B, identification codes 6c, 9c, and 21b).

Correlations between disruption of implicit knowledge acquisition and motor impairments by patients at different stages of disease were reported by Vandenbossche et al. (2009) on patients receiving dopaminergic medication (Figure 1B, 19c), by Muslimovic et al. (2007) on patients with and without dopaminergic medication (Figure 1B, 14d), and by Pavão et al. (unpublished), on patients tested without the effects of dopaminergic medication (Figure 1B, identification code 15c; see Table S1 in Supplementary Material).

### Cognitive and motor impairments

Figure 1C expresses a rapid progression of cognitive impairments associated with a much slower progression of motor impairments at earlier stages of the disease, followed by acceleration of motor impairments at later stages of the disease associated with the cognitive function already deteriorated. As seen above, while dopaminergic medication reduces motor impairments associated with the disease progression, it slightly increases cognitive impairments.

Muslimovic et al. (2007) showed that PD patients exhibited cognitive impairments in absence of motor impairments (Figure 1C, identification codes 14c and 14h; see Table S1 in Supplementary Material) when tested at early stages of the disease without the effects of dopaminergic medication. However, the combined analysis of their performances and the performances observed in patients at advanced stages of the disease tested under the effects of dopaminergic medication showed substantial cognitive and motor impairments (Figure 1C, 14b and 14g).

In addition, significant correlations between cognitive and motor impairments were found in early to advanced (Fama and Sullivan, 2002; Vandenbossche et al., 2009; Verbaan et al., 2007) and advanced PD patients (Caviness et al., 2007) tested under the effects of dopaminergic medication (Figure 1C, identification codes, 6a, 19b, 20a, and 1). Similarly, significant correlations between cognitive and motor impairments were found in patients tested without the effects of dopaminergic medication (identification code 15b; Pavão et al., unpublished) and pooled early to advanced patients with and without dopaminergic medication [identification code 13; Mortimer et al., 1982). Less consistent correlation between cognitive and motor impairments in patients at early stages of the disease tested without the effects of dopaminergic medication were reported by Cooper et al. (1991), which showed both significant and non-significant correlations (Figure 1C, identification codes 3a and 3b), likely related to the characteristics of the employed tasks.

The remaining comparisons involving medicated PD, non-medicated PD and control subjects, and the medication effects in healthy subjects indicated in Figure 1C (identification codes 2a, 3c, 3d, 4, 6b, 6c, 8, 9b, 9d, 10a, 10b, 11, 12, 16, 18, 20b, and 21b) were already analyzed above.

### Disruption of implicit knowledge acquisition, motor impairment and cognitive impairments, and the effects of dopaminergic medication

Figure 1D represents a unified model expressing the interaction between disruption of implicit knowledge acquisition, motor impairments, and general cognitive impairments, in patients at early to advanced stages of the disease, tested both with and without the effects of dopaminergic medication, along the disease progression. As shown, the progression of PD manifestations follows different time courses, and dopaminergic medication has different effects on each of these functions according to disease stages.

## Discussion

Assuming that progressions of motor impairments, disruption of implicit knowledge acquisition, and cognitive impairments in PD follow non-linear relationships (Figure 1), the present model provides an explanation for the apparent conflict of data from different laboratories and for the effects of dopaminergic medication on these manifestations (see Cooper et al., 1991; Muslimovic et al., 2007; Vandenbossche et al., 2009; Pavão et al., unpublished).

Shortly, general cognitive impairments seem to be pronounced at early stages of PD reaching a plateau when disruption of implicit knowledge acquisition, paralleled by motor impairments, are still in progression (Cooper et al., 1991; Fama and Sullivan, 2002; Fern-Pollak et al., 2004; Deroost et al., 2006; Muslimovic et al., 2007; Vandenbossche et al., 2009). Additionally, at any stage of PD, dopaminergic medication seems to promote a substantial reduction of motor impairments, associated with increased disruption of implicit knowledge acquisition (Swainson et al., 2000; Fern-Pollak et al., 2004; Jahanshahi et al., 2010; Kwak et al., 2010; Seo et al., 2010; Domellöf et al., 2011), and a subtle, if any, further impairment of cognition (Press et al., 2002; Feigin et al., 2003; Fern-Pollak et al., 2004; Morrison et al., 2004).

In addition to explaining the apparent conflict of data from different laboratories, this model may be extended. For instance, development of depression, a relevant aspect of the PD that correlates with motor impairments (Cooper et al., 1991), has not been considered here, and could also give rise to apparent conflicting results. An interesting challenge would be to integrate this model with those advanced by Cools (2006) and Rowe et al. (2008) distinguishing executive functions differentially affected by PD.

Independently on these possible future steps, the present model seems to be useful for understanding the multifaceted progression of PD and has testable predictive value regarding the progression of PD manifestations as influenced or not on dopaminergic therapy.

## Conflict of Interest Statement

The authors declare that the research was conducted in the absence of any commercial or financial relationships that could be construed as a potential conflict of interest.

## Supplementary Material

The Supplementary Material for this article can be found online at: http://www.frontiersin.org/Integrative_Neuroscience/10.3389/fnint.2012.00056/abstract

Supplementary Table S1 **The major findings of multiple studies were classified into three functional domains, including motor, implicit knowledge acquisition and general cognitive functions**. The disease stage at which the patients were tested, presence of dopaminergic medication effects on test results, tasks employed, major results observed and correlation analysis with other functional dimensions (when evaluated) were also included.
