# Supplementary material for: Parkinson’s Disease Progression: Implicit Acquisition, Cognitive and Motor Impairments, and Medication Effects
Source: Front Integr Neurosci. 2012 Aug 10;6:56. doi: 10.3389/fnint.2012.00056 (PMC3415726; doi:10.3389/fnint.2012.00056)
Supplement: Supplementary Table S1 — The major findings of multiple studies were classified into three functional domains, including motor, implicit knowledge acquisition and general cognitive functions. The disease stage at which the patients were tested, presence of dopaminergic medication effects on test results, tasks employed, major results observed and correlation analysis with other functional dimensions (when evaluated) were also included. [file 29101_Pav__o_DataSheet1.PDF]

Function	Reference	Population	Medication	Tasks	Identification code	Findings
Cognitive	Caviness et al. (2007)	Advanced PD *(75±8.5 y. old, 6.3±4.7 y. disease divided in two subgroups)*	*yes (levodopa equivalents 515±387 mg)*	Rey auditory verbal learning test delayed, Controlled oral word association , Verbal Fluency, Stroop Color and Word Test **(see the motor tasks employed in this study in the corresponding sector of this table, below)**	**1**	**Medicated advanced PD patients: cognition correlated with motor** (subgroup with worse scores in Rey auditory learning test delayed and Stroop color and word test exhibited better scores UPDRS-motor, suggesting that cognition and motor functions parallel to each other)
	Cools et al. (1984)	Healthy *(60±11.6 y. old)* and early to advanced PD *(60.8±9.3y. old, 5.8±3.1 y. disease, 1.5-14.5)*	yes (early to advanced *(88.9%of the sample), taking levodopa. carbidopa, benserazide, orfenadrine, dexetinide, benzatropine and/or amantadine)* / no (early to advanced *(11.1%)* and healthy)	Word production (animals/professions), sorting blocks (form/color/size) and animals (bird-mammal/domestic-wild)	**2a**	**Unmedicated/medicated early to advanced PD patients: cognition poorer than that seen in unmedicated healthy subjects** (patients produced fewer different names and needed more trials for detecting a shift in a sorting criterion than healthy subjects)
	Cooper et al. (1991)	Healthy *(59.6 y. old, 40.2-76.1)* and early PD *(58.5 y. old, 40.2-76.1; 1.3 y. disease, 0.25-4)*	no *(untreated)*	WMS (mental control, logical memory digit span forward and backward, visual reproduction, associate learning), WAIS (vocabulary), Digit ordering, Picture arrangement, Language (Boston Naming, Token, Reporter's and Semantic Fluency tests), Rey Osterriech and Taylor complex figures (copy and recall), Brown Peterson Distractor test (immediate and delayed), WCST, Golling incomplete figures (immediate and delayed), Matchsticks test, Blessed dementia scale **(see the motor tasks employed in this study in the corresponding sector of this table, below)**	**3a**	**Unmedicated early PD patients: cognition correlated with motor function** (WMS digit span backward and visual reproduction, recall of the Rey Osterriech and Taylor complex figures, the Picture arrangement and delayed Golling incomplete figures scores correlate with motor scores in the Fine Finger Movements Test. WMS visual reproduction and copy of the Rey Osterriech and Taylor complex figures scores correlated with motor scores in KCRS. PD patients exhibiting scores in the normal range in the Blessed dementia scale presented better motor scores in the Fine Finger Movements Test than PD patients exhibiting scores below the normal range in the Blessed dementia scale, suggesting that cognition and implicit acquisition parallel to each other.
					**3b**	**Unmedicated early PD patients: cognition did not correlate with motor function** (WMS mental control, logical memory digit span forward and associate learning, WAIS vocabulary, Digit ordering, Language Boston Naming, Token, Reporter's and Semantic Fluency tests, copy of Rey Osterriech and Taylor complex figures, Brown Peterson Distractor test, WCST, immediate Golling incomplete figures, Matchsticks test and Blessed dementia scale did not correlate with motor scores in the Fine Finger Movements Test. WMS mental control, logical memory digit span forward and backward, and associate learning, WAIS vocabulary, Digit ordering, Picture arrangement, Language Boston Naming, Token, Reporter's and Semantic Fluency tests, recall of Rey Osterriech and Taylor complex figures, Brown Peterson Distractor test, WCST, Golling incomplete figures, Matchsticks test and Blessed dementia scale did not correlate with motor scores in KCRS)
					**3c**	**Unmedicated early PD patients: cognition poorer than that seen in unmedicated healthy subjects** (WMS mental control, logical memory digit span backward, visual reproduction and associate learning, Digit ordering, Picture arrangement, Language Boston Naming, Token, Reporter's and Semantic Fluency tests, copy of Rey Osterriech and Taylor complex figures, delayed Brown Peterson Distractor test, WCST and Blessed dementia scale)
	Delaveau et al. (2005)	Healthy *(55.6±6.8 y. old)*	yes *(100mg levodopa)* / no *(placebo)*	Emotional Facial Perception Test	**4**	**Medicated healthy subjects: cognition poorer than that seen in unmedicated healthy subjects**
	Fama and Sullivan (2002)	Healthy *(61.2±7.9 y. old, 48-74)* and early to advanced PD *(62.4±7.3 y. old, 48-75; 6.7±6.4 y. disease, 1-29)*	yes (PD, *standard anti-parkinsonism medication*) / no (healthy)	WAIS Picture Arrangement subtest, WCST categories **(see the motor tasks employed in this study in the corresponding sector of this table, below)**	**6a**	**Medicated early to advanced PD patients: cognition correlated with motor function** (the Picture Arrangement scores correlated with the Fist-Ring and Fist-Edge-Palm scores; the WCST categories scores correlated with scores of all motor tasks)
					**6b**	**Medicated early to advanced PD patients: cognition was poorer than that seen in unmedicated healthy subjects** (both cognitive tasks)
	Girotti et al. (1986)	Healthy *(57.8±7 y. old)* and advanced PD *(58±8.1 y. old; 11±4.8 y. disease)*	yes (advanced PD: levodopa, *904±275.5 mg, and carboxilase inhibitor; in some of patients, also bromocriptine, 25.6±17.4 mg, or lisurine 4±1.5 mg)* / no (advanced PD*: withdrawal;* healthy*: untreated*)	Set test (word recall), Randt memory test, Rene Zazzo's attention test	**9a**	**Medicated advanced PD patients: cognition was poorer than that seen in unmedicated healthy subjects**
					**9b**	**Unmedicated advanced PD patients: cognition did not differ relative to that seen in medicated advanced PD patients**
	Growdon et al. (1998)	Early to advanced PD *(63.7±9.7 y. old, 3.9±1.6 y. disease)*	yes *(≤6 months of levodopa 3.9±1.8mg/kg/day; carbidopa);* no *(pre-treatment)*	Digit Span, Digit Symbol, Selective Reminding, Odd Man Out, New Dot Tests and Verbal Fluency tests	**10a**	**Unmedicated early to advanced PD patients: cognition did not differ relative to that seen in medicated early to advanced PD patients** (all tasks, except Digit Symbol, Odd Man Out, and Verbal Fluency tests, subtly better (but statistically significant) in the drug-on condition)
	Morrison et al. (2004)	Advanced PD *(65.1±7.1 y. old; 10.3±3.3 y. disease*)	yes *(levodopa)* / no *(withdrawal of 10–15h)*	Randt Memory Test, Brief Test of Attention, Hopkins Verbal Learning Test-R, Boston Naming Test, Verbal Fluency, Visual Form Discrimination Test, Judgment of Line Orientation Test, Standardized Test of Direction Sense, Odd Man Out Test, Stroop Color and Word Test, Alternating Verbal Fluency, Geriatric Depression Scale	**12**	**Unmedicated advanced PD patients: cognition did not differ relative to that seen in medicated advanced PD patients** (all tasks, except Boston Naming Test, better in the drug-off condition)
	Mortimer et al. (1982)	Early to advanced PD *(62.67±8.22 y. old 9.35±8.14 y. disease)*	yes *(levodopa or sinemet (88.6% of the sample))* / no *(11.4%)*	WAIS block design and digit symbol subtests; visual discrimination test **(see the motor tasks employed in this study in the corresponding sector of this table, below)**	**13**	**Unmedicated and medicated early to advanced PD patients: cognition correlated with motor function** (WAIS block design and digit symbol subtests; visual discrimination test correlated with the bradykinesia test)
	Muslimovic et al. (2007)	Healthy *(64.1±8.3 y. old (n=44))*, early PD *(60.7±10.8 y. old; 1.2±0.5 y. disease (n=24)*, and early to advanced PD *(64.9±8.9 y. old; 3.1±2.6 y. disease, (n=95, wich includes the ealy PD group))*	yes *(advanced PD, levodopa/peripheral levodopa-decarboxylase inhibitor (39.4% of the sample), pergolide (12.7%), pramipexol (5.6%), ropinirol (2.8%), levodopa/pergolide (22.5%), levodopa/pramipexol (2.8%), levodopa/ropinirol (1.4%), amantadine (2.8%), amantadine/orfenadrine (1.4%), levodopa/amantadine (1.4%), levodopa/entacapone (4.2%), levodopa/orfenadrine (1.4%), levodopa/pergolide/entacapone (1.4%)*) / no *(healthy and early PD)*	Attention and psychomotor speed (WAIS digit symbol test, Trail Making Tests A and B, Stroop test - word reading, color naming and interference tests, and Digit span - forward and backward), Memory (RAVLT - trial 1 to 5, delayed recall and recognition, RBMT - immediate and delayed recall, WMS faces - immediate and delayed, and Visual Association Test), Executive functions (WCST - categories and perseveration, Fluency - animals and supermarket, COWAT letter fluency, Tower of London test, and WAIS similarities) and Visuospatial/constructive skills (JOLO, GIT visuospatial reasoning, and Clock Drawing Test) **(see the implicit acquisition and motor tasks employed in this study in the corresponding sector of this table, below)**	**14a**	**Unmedicated/medicated early to advanced PD patients: cognition did not correlate with implicit knowledge acquisition** (global analysis including the z-scores of Attention and psychomotor speed, Memory, Executive functions and Visuospatial/constructive skills domains did not correlate with scores in SRT - sequence learning)
					**14b**	**Unmedicated/medicated early to advanced PD patients: cognition was poorer than that seen in unmedicated healthy subjects** (global analysis showed impairments of Attention and psychomotor speed, Memory, Executive functions and Visuospatial/constructive skills domains. However, there were no significant differences in the Stroop test - word reading and color naming, Digit span – forward, RAVLT – recognition, Visual Association Test, Animal fluency, COWAT letter fluency, WAIS similarities and Clock Drawing Test)
					**14c**	**Unmedicated early PD patients: cognition was poorer than that seen in unmedicated healthy subjects** (Digit Symbol test, faces immediate recognition test and Tower of London test)
	Pavão et al. (unpublished)	Early to advanced PD *(64.1±9.8 y. old, 45-81; 1.9±2.2 y. disease, 0-6)*	no *(nonmedicated (53% of the sample), >8h of withdrawal of levodopa (29.4%), amantadine (17.6%))*	Reaction times (RT) and error rate (ER) on Go/no-go (GNG) and 1-back working memory (1BK) tests with low frequency of active responses **(see the implicit acquisition and motor tasks employed in this study in the corresponding sector of this table, below)**	**15a**	**Unmedicated early to advanced PD patients: cognition correlated with implicit acquisition** (GNG-RT correlated with SRT-SA)
					**15b**	**Unmedicated early to advanced PD patients: cognition correlated with motor function** (GNG-RT correlated with SRT-MP and SiRT-RT; GNG-ER correlated with SiRT-RT; 1BK-ER correlated with SRT-MP and SiRT-RT.
	Sabbe et al. (2004)	Healthy (31.6± 8.7 y. old)	yes *(250 mg levodopa)* / no *(placebo)*	Figure copying - time on planning	**16**	**Medicated healthy subjects: cognition was poorer than that seen in unmedicated healthy subjects**
	Vandenbossche et al. (2009)	Early to advanced PD *(64.8±4.7 y. old; 8.6±2.9 y. disease)*	yes *(levodopa/dopamine agonists (88% of the sample), levodopa (8%), dopamine agonists (4%))*	SCOPA-COG (Memory and learning, Attention, Executive functions, Visuospatial functions) **(see the implicit acquisition and motor tasks employed in this study in the corresponding sector of this table, below)**	**19a**	**Medicated early to advanced PD patients: cognition correlated with implicit acquisition** (subjects exhibiting scores within the normal range in the SCOPA-COG exhibited better scores in SRT - sequence learning, suggesting that cognition and implicit acquisition parallel to each other)
					**19b**	**Medicated early to advanced PD patients: cognition correlated with motor function** (subjects exhibiting scores within the normal range in the SCOPA-COG exhibited better scores in SRT - reaction time in random sequence blocks, suggesting that cognition and motor performance parallel to each other)
	Verbaan et al. (2007)	Healthy *(61±12.4 y. old)* and early to advanced PD *(60.8±11.4 y. old; 10.5±6.4 y. disease)*	yes (patients *treated with levodopa 277±69mg, dopamine agonist and/or cholinesterase inhibitors (95.8% of the sample); not treated (4.2%))* / no (healthy)	SCOPA-COG (Memory and learning, Attention, Executive functions, Visuospatial functions)**(see the motor tasks employed in this study in the corresponding sector of this table, below)**	**20a**	**Medicated early to advanced PD patients: cognition correlated with motor** (higher scores in SCOPA-COG quartiles associated to higher scores in SPES-SCOPA-motor, suggesting that cognition and motor functions parallel to each other)
					**20b**	**Medicated early to advanced PD: cognition poorer than that seen in unmedicated healthy subjects** (patients exhibited worse scores in global scale and in each sub-domain in comparison to healthy subjects)

**Implicit acquisi-tion**	Cools et al. (1984)	Early to advanced PD *(see details in cognitive sector of this table)*	yes (early to advanced) / no (early to advanced and healthy) *(see details in cognitive sector of this table)*	Press buttons in two given sequences	**2b**	**Unmedicated/medicated early to advanced PD patients: implicit acquisition was poorer than that seen in unmedicated healthy subjects** (patients produced fewer finger responses in both sequences in comparison to healthy subjects)
	de Vries et al. (2010)	Healthy *(24.2± 3.1 y. old)*	yes *(100 mg levodopa, 25 mg carbidopa)* / no *(placebo)*	Artificial grammar learning	**5**	**Medicated healthy subjects: implicit acquisition was better than that seen in unmedicated healthy subjects**
	Floel et al. (2005)	Healthy *(47±18.2 y. old, 24 –87)*	yes *(100 mg levodopa, 25 mg carbidopa)* / no *(placebo)*	Transcranial magnetic stimulation evoked thumb movement directions (frequency of movements in the trained direction)	**7**	**Medicated healthy subjects: implicit acquisition was better than that seen in unmedicated healthy subjects**
	Muslimovic et al. (2007)	Healthy, early PD, and early to advanced PD *(see details in cognitive sector of this table)*	yes (advanced PD) / no (healthy and early PD) *(see details in cognitive sector of this table)*	SRT - sequence learning **(see the cognitive and motor tasks employed in this study in the corresponding sector of this table)**	**14a**	**Unmedicated/medicated early to advanced PD patients: implicit acquisition did not correlate with cognition**
					**14d**	**Unmedicated/medicated early to advanced PD patients: implicit acquisition correlated with motor function** (early unmedicated PD patients exhibited better sequence learning scores and lower reaction times when compared to pooled data including early unmedicated and advanced medicated PD patients, suggesting that implicit acquisition and motor performance parallel to each other)
					**14e**	**Unmedicated/medicated early to advanced PD patients: implicit acquisition was poorer than that seen in unmedicated healthy subjects**
					**14f**	**Unmedicated early PD patients: implicit acquisition was similar to that seen in unmedicated healthy subjects**
	Pavão et al. (unpublished)	Early to advanced PD *(see details in cognitive sector of this table)*	no *(see details in cognitive sector of this table)*	Sequence acquisition score (SA) in the Finger-to-thumb SRT **(see the cognitive and motor tasks employed in this study in the corresponding sector of this table)**	**15a**	**Unmedicated early to advanced PD patients: implicit acquisition correlated with cognition** (SRT-SA correlated with GNG-RT)
					**15c**	**Unmedicated early to advanced PD patients: implicit acquisition correlated with motor function** (SRT-SA correlated with SRT-MP)
	Seo et al. (2010)	Healthy *(66±1.9 y. old)* and advanced PD *(med.: 67±1.8 y. old; 13.5±2.2 y. disease; unmed: 68±2.5 y. old; 11.5±2.3 y. disease)*	yes (advanced PD, *levodopa*) / no (healthy, *unmedicated*, and advanced PD, *14.65±1.59h of levodopa withdrawal*)	Sequence learning task with stochastic feedback	**17a**	**Unmedicated advanced PD patients: implicit acquisition was poorer than that seen in unmedicated healthy subjects**
					**17b**	**Medicated advanced PD patients: implicit acquisition was poorer than that seen in unmedicated healthy subjects**
					**17c**	**Unmedicated advanced PD patients: implicit acquisition was better than that seen in medicated advanced PD patients**
	Vandenbossche et al. (2009)	Early to advanced PD *(see details in cognitive sector of this table)*	yes *(see details in cognitive sector of this table)*	SRT - sequence learning **(see the cognitive and motor tasks employed in this study in the corresponding sector of this table)**	**19a**	**Medicated early to advanced PD patients: implicit acquisition correlated with cognition** (see 19a-cognition, above)
					**19c**	**Medicated early to advanced PD patients: implicit acquisition correlated with motor function** (subjects exhibiting higher sequence learning scores exhibited had higher motor scores, suggesting that implicit acquisition and motor performance parallel to each other)
	Wilkinson and Jahanshahi (2007)	Advanced PD *(62.8±8.1 y. old, 45-73; 9.27 ±94.92 y. disease)* and healthy *(56±10.3 y. old, 43-72)*	no (advanced PD: *withdrawal of levodopa for 13.6±3.8h*; healthy: *unmedicated*)	SRT – sequence learning	**21a**	**Unmedicated advanced PD patients: implicit acquisition was poorer than that seen in unmedicated healthy subjects**
**Motor**	Caviness et al. (2007)	Advanced PD *(see details in cognitive sector of this table)*	yes *(see details in cognitive sector of this table)*	UPDRS (motor examination) **(see the cognitive tasks employed in this study in the corresponding sector of this table, above)**	**1**	**Medicated advanced PD patients: motor function correlated with cognition** (see 1-cognition, above)
	Cooper et al. (1991)	Healthy and Early PD *(see details in cognitive sector of this table)*	no *(see details in cognitive sector of this table)*	Fine Finger Movements test and the King's College Rating Scale (KCRS) **(see the cognitive tasks employed in this study in the corresponding sector of this table, above)**	**3a**	**Unmedicated early PD patients: motor function correlated with cognition** (see 3a-cognition, above)
					**3b**	**Unmedicated early PD patients: motor function did not correlate with cognition** (see 3b-cognition, above)
					**3d**	**Unmedicated early PD patients: motor function was poorer than that seen in unmedicated healthy subjects** (in the Fine Movement test. Note that the healthy subjects were not tested in the KCRS)
	Fama and Sullivan (2002)	Healthy and early to advanced PD *(see details in cognitive sector of this table)*	yes (PD) / no (healthy) *(see details in cognitive sector of this table)*	Fine Finger Movements Test (motor rigity), Motor Sequencing, Ozeretski Test (motor sequencing), Fist-Ring Test (motor sequencing), Pronation-Supination Test, Fist-Edge-Palm Test (motor sequencing) **(see the cognitive tasks employed in this study in the corresponding sector of this table, above)**	**6a**	**Medicated early to advanced PD patients: motor function correlated with cognition** (see IC_Fama_2004_a-cognition, above)
					**6c**	**Medicated early to advanced PD patients: motor function was poorer than that seen in healthy subjects** (all tests, except Ozeretski Test)
	Floel et al., 2008	Healthy *(47±19.3 y. old, 21-70)*	yes *(100 mg levodopa, 25 mg carbidopa)* / no *(placebo)*	Jebsen-Taylor test of hand functions	**8**	**Medicated healthy subjects: motor function was better than that seen in unmedicated healthy subjects**
	Girotti et al. (1986)	Healthy and advanced PD *(see details in cognitive sector of this table)*	yes (advanced PD) / no (advanced PD and healthy) *(see details in cognitive sector of this table)*	Reaction time and movement time of predicted and unpredicted stimulus	**9c**	**Medicated advanced PD patients: motor function was poorer than that seen in healthy subjects** (patients presented higher movement times than healthy)
					**9d**	**Unmedicated advanced PD patients: motor function was poorer than that seen in medicated advanced PD patients** (unmedicated patients presented higher movement times than medicated patients)
	Growdon et al. (1998)	Early to advanced PD *(see details in cognitive sector of this table)*	yes / no *(see details in cognitive sector of this table)*	UPDRS (motor examination)	**10b**	**Medicated early to advanced PD patients: motor function was better than in unmedicated early to advanced PD patients**
	Hasbroucq et al. (2003)	Healthy *(mean 25 y. old, 21–28)*	yes *(200 mg levodopa, 25 mg benserazide,)* / no *(placebo)*	Choice RT and movement time	**11**	**Medicated healthy subjects: motor function was better than in unmedicated healthy subjects** (both motor tasks)
	Mortimer et al. (1982)	Early to advanced PD *(see details in cognitive sector of this table)*	yes / no *(see details in cognitive sector of this table)*	Bradykinesia test: covering a randomly moving spot on a cathode ray display with a photodetector held with the outstretched hand and to do this as rapidly as possible (a "hit" was scored for each coincidence of detector and spot.) **(see the cognitive tasks employed in this study in the corresponding sector of this table, above)**	**13**	**Unmedicated and medicated early to advanced PD patients: cognition correlated with motor function** (see 13-cognition, above)
	Muslimovic et al. (2007)	Healthy, early PD, and early to advanced PD *(see details in cognitive sector of this table)*	yes (advanced PD) / no (healthy and early PD) *(see details in cognitive sector of this table)*	SRT – reaction time **(see the cognitive and implicit acquisition tasks employed in this study in the corresponding sector of this table, above)**	**14d**	**Unmedicated/medicated early to advanced PD patients: motor function correlated with implicit acquisition** (see 14d-implicit acquisition, above)
					**14g**	**Unmedicated/medicated early to advanced PD patients: motor function was poorer than that seen in healthy subjects**
					**14h**	**Unmedicated early PD patients: motor function did not differ from that seen in healthy subjects**
	Pavão et al. (unpublished)	Early to advanced PD *(see details in cognitive sector of this table)*	no *(see details in cognitive sector of this table)*	Reaction time (RT) on Finger-to-thumb opposition SRT and Simple Reaction Time (SiRT) tasks **(see the cognitive and implicit acquisition tasks employed in this study in the corresponding sector of this table, above)**	**15b**	**Unmedicated early to advanced PD patients: motor function correlated with cognition** (see 15b-cognition, above)
					**15c**	**Unmedicated early to advanced PD patients: motor function correlated with implicit acquisition** (see 15c-implicit acquisition, above)
	Stocchi et al. (2005)	Advanced PD *(63.2±11.1 y. old, 48-81; 17.2±7.2 y. disease, 11-28)*	yes *(levodopa, 1233.3±98.3 mg)* / no *(withdrawal)*	UPDRS (motor examination)	**18**	**Unmedicated advanced PD patients: motor function was poorer than that seen in medicated advanced PD patients**
	Vandenbossche et al. (2009)	Early to advanced PD *(see details in cognitive sector of this table)*	yes *(see details in cognitive sector of this table)*	SRT - reaction time on random sequence blocks **(see the cognitive and implicit acquisition tasks employed in this study in the corresponding sector of this table, above)**	**19b**	**Medicated early to advanced PD patients: motor function correlated with cognition** (see 19b-cognition, above)
					**19c**	**Medicated early to advanced PD patients: motor function correlated with implicit acquisition** (see 19c-implicit acquisition, above)
	Verbaan et al. (2007)	Early to advanced PD *(see details in cognitive sector of this table)*	yes *(see details in cognitive sector of this table)*	SPES-SCOPA-motor **(see the cognitive tasks employed in this study in the corresponding sector of this table, above)**	**20a**	**Medicated early to advanced PD patients: motor function correlated with cognition** (see 20a-cognition, above)
	Wilkinson and Jahanshahi (2007)	Advanced PD and healthy *(see details in implicit acquisition sector of this table)*	no *(see details in implicit acquisition sector of this table)*	SRT – reaction time	**21b**	**Unmedicated advanced PD patients: motor function was poorer than that seen in unmedicated healthy subjects**
